# Does increased local bone resorption secondary to breast and prostate cancer result in increased cartilage degradation?

**DOI:** 10.1186/1471-2407-8-180

**Published:** 2008-06-27

**Authors:** Diana J Leeming, Inger Byrjalsen, Per Qvist, Mitsuru Koizumi, Niels Lynnerup, Michael Fregerslev, Mette G Sørensen, Claus Christiansen, Morten A Karsdal

**Affiliations:** 1 Nordic Bioscience, Herlev Hovedgade 207, 2730 Herlev, Denmark; 2 National Institutes of Radiological Sciences, Anagawa 4-9-1, Chiba, Japan; 3 Institute of Forensic Medicine, University of Copenhagen, The Panum Institute, Blegdamsvej 3, DK-2200, Copenhagen, Denmark

## Abstract

**Background:**

Breast and prostate cancer patients often develop lesions of locally high bone turnover, when the primary tumor metastasizes to the bone causing an abnormal high bone resorption at this site. The objective of the present study was to determine whether local increased bone turnover in breast and prostate cancer patients is associated with an increase in cartilage degradation and to test *in vitro *whether osteoclasts or cathepsin K alone generate CTXII from human bone.

**Methods:**

The study included 132 breast and prostate cancer patient, where presence of bone metastases was graded according to the Soloway score. Total bone resorption (CTXI_total_) and cartilage degradation (CTXII) were determined.

**Results:**

Breast and prostate cancer patients with bone metastases revealed significant increased levels of CTXI_total _at Soloway scores 1 and higher compared to patients without bone metastases (p < 0.001). CTXII was statistically elevated at score 3 and 4 (p < 0.01). CTXII/CTXI_total _significantly decreased at score 3 and 4 (p < 0.001). Levels of CTXI_total_, CTXII and CTXII/CTXI_total _changed +900%, +130%, and -90%, respectively at Soloway score 4 compared to score 0. The *in vitro *experiments revealed that osteoclasts released CTXI fragments but not CTXII from bone specimens. The same was observed for cathepsin K.

**Conclusion:**

Data suggest that an uncoupling between bone resorption and cartilage degradation occurs in breast and lung cancer patient.

## Background

Bone metastases are common in breast and prostate cancer patients causing an abnormal high bone remodeling at these sites of metastasis destroying the bone structure. Bone metastases occur in more than 50% of these patients with advanced disease [1,2]. The pathology of bone metastasis is characterized by a vicious cycle where the equilibrium between the bone resorbing cells "osteoclasts" and the bone forming cells "osteoblasts" is unbalanced causing highly elevated activity and number of cells [2].

Bone and cartilage degradation can be measured in serum and urine samples by biochemical markers such as bone resorption by CTXI [3] and cartilage degradation by CTXII [4]. These markers have been used extensively for the evaluation of these parameters in basic, pre-clinical and clinical studies [5].

Anti-resorptive drugs have been shown to cause a decrease in both cartilage degradation and bone resorption both in postmenopausal women and Paget's disease patients [6-8] indicating a coupling between the two processes. Type II collagen has been identified in both the metaphysis and diaphysis of rats [9], which was thought to be exclusively expressed in cartilage, suggesting that part of the cartilage degradation level could originate from the type II collagen content in bone.

The aim of this study was to resolve whether the coupling that has been observed between cartilage degradation and bone resorption, is coupled in bone metastatic cancer characterized by local high bone turnover. We have previously assessed eight different biochemical markers in a group of breast, lung and prostate cancer patients [10]. In the present study we compared the levels of cartilage degradation as measured by CTXII to the levels of total bone resorption (total CTXI) in a subset of this cohort. Additionally, we wanted to investigate whether CTXII could be released from human bone specimens by osteoclasts *in vitro*, bearing in mind that CTXII is present in the rat bone. As the CTXII epitope might be destroyed by other enzymatic activities exhibited by the osteoclast we additionally investigated the degradation of human bone by cathepsin K alone.

## Methods

### Patients and study design

The cohort including a set of lung cancer patients has been described elsewhere [10]. A total of 132 breast and prostate cancer patients were referred to the Cancer Institute Hospital, Tokyo between October 2002 and April 2004. All patients underwent bone scanning using Technetium-99 m scintigraphy together with computer tomography and/or magnetic resonance imaging to verify and quantify the presence of bone metastases (BM). The skeletal load was graded, according to [11]. Soloway 0 refers to patients without BM, Soloway 1 to patients with less than 6 BM, Soloway 2 to patients with less than 20 BM, Soloway 3 to patients with more than 20 but less than a "super scan", Soloway 4 to patients with a "super scan" that is defined by a more than 75% involvement of the ribs, vertebrae, and pelvic bones.

Second void morning urine was collected from each patient and stored at -40°C until assaying. All patients with skeletal complications were newly diagnosed and none had received therapies known to influence bone turnover in the past 2 years prior to entry to the study. All participants signed an approved written consent; the study was done in accordance to the Helsinki Declaration II and Standards of Good Clinical Practice. The Local Ethical Committee had approved the study protocol.

### Quantification of biochemical markers in the clinical study

Urine was used for estimation of bone resorption by measuring the level of total CTXI (CTXI_total)_. The level of cartilage degradation was assessed by measurement of CTXII levels. The concentration of alpha CTX and beta CTX fragments was measured by the urinary ALPHA CrossLaps [12] and serum CrossLaps (urine samples were diluted 1:25), respectively. Total CTXI was calculated as the sum of alpha and beta CTXI. All assays were from Nordic Bioscience, Herlev, Denmark and run according to manufacturer's instructions. The ratio between CTXII and total CTXI was calculated as CTXII/CTXI_total_.

### Human bone samples

Human cortical bones were collected during autopsy at the Forensic Science Institute, Copenhagen, Denmark and stored in 70% EtOH at 4°C. 2 cm long bone sticks from one individual, were drilled from the central part of femoral midshafts specimens. A stick was cut into 0.2 mm thick slices with a diameter that fits into 96-well culture plates. The Danish Ethical Committee had approved the study (study no. KF01295491).

### Human osteoclast precursors and differentiation on human cortical bone

The isolation of CD14+ monocytes was performed as previously described [13]. Briefly, human monocytes were isolated from peripheral blood by centrifugation on a Ficoll-Paque gradient (Amersham Pharmacia), and magnetically sorted using a CD14+ magnetic bead isolation kit (Dynabeads M-450, Dynal Biotech). The cells were then seeded in 75 cm^2 ^flasks, and cultured in αMEM containing 10% fetal calf serum, 100 U/mL penicillin, 100μg/mL streptomycin, and 25 ng/ml of macrophage-colony stimulating factor (M-CSF) (R&D Systems) for 3 days, then they were lifted, re-seeded and cultured on human cortical bone slices in the presence of 25 ng/ml M-CSF and 25 ng/ml receptor activator of NF-κB ligand (RANKL) (R&D Systems) for 28 days. Conditioned medium was collected every second or third day.

### Cathepsin K degradation of human cortical bone

Human cortical bone slices were decalcified for 4 days at 4°C by ethylenediamine tetraacetic acid (EDTA) (0.5 mol/L EDTA, 0.05 mol/L Tris, 1% NaN_3_, pH 7.5) and washed in MilliQ-water. Bone slices of 4.4 mg were snap-frozen in liquid nitrogen and pulverized using a Bessman tissue pulverizer (Spectrum, The Netherlands). 750 μg bone powder was transferred to eppendorfh tubes and mixed with 650 μL cathepsin K buffer (50 mM Na-Acetat, 20 mM L-Cystine, pH 5.5), homogenized 1/2–1 min. using a Polytron homogenizer at speed level 5. Recombinant human cathepsin K (Calbiochem, cat. no. 342001) was activated as recommended by manufacturer and 17 μL 100 μg/mL activated cathepsin K or control was added to 100 μL bone powder mixture. Samples were incubated overnight at 37°C and centrifuged at 1700 × *g *for 10 minutes; supernatants were harvested and centrifuged again at 10,000 × *g*.

### Quantification of biochemical markers in the in vitro studies

Levels of CTXI and CTXII fragments were assessed in the conditioned medium using CrossLaps for culture (beta CTX) and urinary Pre-Clinical CartiLaps (CTXII), respectively. Both were from Nordic Bioscience, Herlev, Denmark and run according to manufacturer's instructions.

### Statistical Analysis

The values of each of the biochemical markers were logarithmically transformed to obtain normality. Comparison of the levels at each Soloway score relative to Soloway score 0 was performed by analysis of variance (ANOVA) using the General Linear Models Procedure of the Statistical Analysis System (SAS, Cary, NC). Differences and associations were considered statistically significant if p < 0.05.

## Results

### Relation to the extent of metastatic bone disease

Demographic data has been published elsewhere [10]. There were no statistically significant differences in age, BMI and sex distribution between the patients with or without BM.

The levels of CTXII, CTXI_total _and CTXII/CTXI_total _were stratified according to the extent of metastatic disease (Soloway score) and data are shown in table 1. CTXI_total _was highly elevated at all Soloway scores (p < 0.001) compared to score 0. CTXII was statistically significantly elevated at Soloway score 3 and 4 (p < 0.01). In contrast, the ratio CTXII/CTXI_total _decreased significantly at Soloway scores 3 and 4 (p < 0.001) compared to score 0. Levels of CTXI_beta _representing the general bone resorption exhibited the same pattern as CTXI_total _with significant elevation at all Soloway scores (score 1: p < 0.05; score 3, 4: p < 0.001) and the ratio CTXII/CTXI_beta _significantly decreased at score 4 (p < 0.001) (data not shown).

**Table 1:** Mean marker levels in cancer patients stratified by Soloway Score

	**Soloway score**				
	
	**0**	**1**	**2**	**3**	**4**
**n**	70	26	13	15	8
**CTXI**_total _**μg/mmol (1 SD range)**	1.65 (0.88–1.88)	2.79*** (1.54–3.41)	3.84*** (1.84–3.54)	6.64*** (3.21–6.22)	16.49*** (7.67–14.35)
**CTXII μg/mmol (1 SD range)**	0.18 (0.09–0.18)	0.23 (0.11–0.19)	0.25 (0.14–0.31)	0.36** (0.22–0.57)	0.41** (0.28–0.85)
**CTXII/CTX**_total _**(1 SD range)**	0.11 (0.05–0.11)	0.08^ns ^(0.04–0.08)	0.07^ns ^(0.04–0.11)	0.05*** (0.04–0.11)	0.03*** (0.02–0.04)
**Cancer type (n)**	44 BC, 26 PC	20 BC, 6 PC	12 BC, 1 PC	12 BC, 3 PC	5 BC, 3 PC

SD = Standard Deviation, BC = Breast Cancer, PC = Prostate Cancer. * indicates significant difference between the Soloway score compared to score 0.

**Figure 1:**
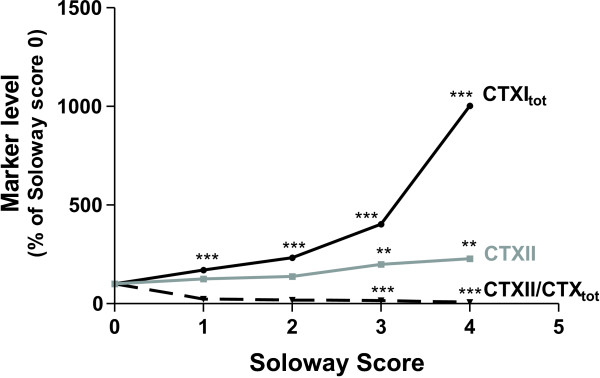
Relative increases in bone and cartilage degradation markers and ratio between these as a function of the extent of skeletal involvement assessed in 132 breast and prostate cancer patients. Relative increases are expressed as percentage of levels in patients with Soloway score 0. Asterisks indicate significant difference compared to the level at score 0.

The level of each marker was calculated relative to the marker level at Soloway score zero. Figure 1 shows the association between Soloway score and markers. It was seen that CTXI_total _clearly increased with extent of metastatic disease; the percentage increase at score 1 was 70% and highest at score 4 presenting a 900% increase. CTXII was elevated at Soloway score 3 with 100% and at score 4 with a 130% increase. The levels of the ratio CTXII/CTXI_total _decreased 50% at score 3 and 80% at score 4. Beta CTXI decreased 60% at score 1 and 620% at score 4 (data not shown)

### Osteoclasts release CTXI but not CTXII from human bone

Human osteoclast precursors were seeded on human bone slices and cultured for 28 days in the presence of RANKL and M-CSF for differentiation and resorption. CTXI and CTXII were assessed in the conditioned medium collected at all time points.

**Figure 2:**
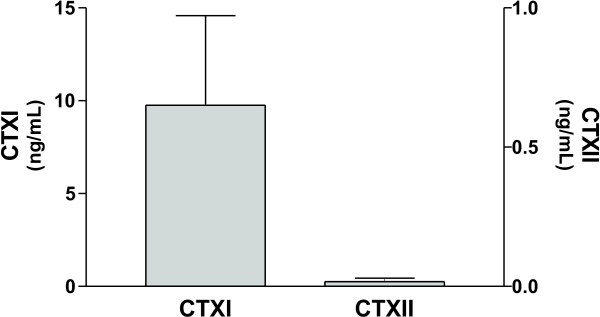
Release of CTXI and CTXII by osteoclastic resorption of human cortical bone at day 28.

During day 1–8 osteoclast precursors had to differentiate into mature osteoclasts thus the level of CTXI and CTXII was zero. After day 8 it was seen that mature osteoclasts on human bone were able to resorb and release CTXI fragments but not CTXII fragments, as previously described on bovine bones [14]. Data on figure 2 shows the level of CTXI and CTXII in the conditioned medium collected at day 28, representing the pattern seen throughout day 9–28.

### Cathepsin K release of CTXI and CTXII from human bone

**Figure 3:**
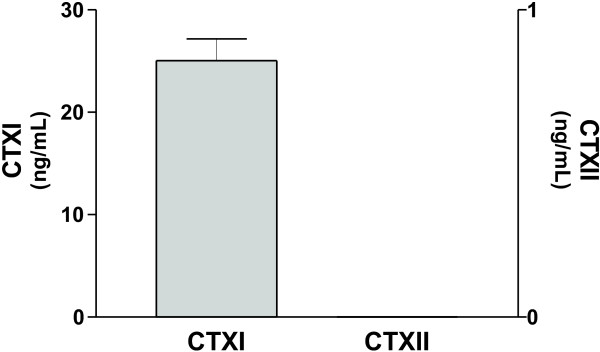
Release of CTXI and CTXII from cathepsin K treated human cortical bone.

From figure 3 it is seen that the release of CTXI was only observed from the positive control; human bone treated with cathepsin K. Release of CTXII fragments by cathepsin K was not observed from bone.

## Discussion

We performed a comparison of bone resorption (CTXI) and cartilage degradation (CTXII) in a cohort of breast and prostate cancer patients stratified by the number of bone metastases to investigate whether the coupling between bone and cartilage degradation observed in postmenopausal women and Paget's patients is uncoupled in these cancer patients. The main findings were as follows: 1) CTXI_total _was significantly increased at all Soloway scores as was expected for a collagenous bone degradation marker, 2) CTXII was not significantly elevated until at Soloway score 3, where progression of the disease had reached to more than 20 bone metastases, 3) The ratio CTXII/CTXI_total _decreased with extent of metastatic disease, showing that the molar ratio of CTXI and CTXII fragments did not follow the same pattern of release through disease progression, 4) Mature osteoclasts resorbing on human cortical bone in an *in vitro *model was not able to CTXII fragments, 5) and cathepsin K degradation of human bone *in vitro *did not result in generation of CTXII fragments.

### Uncoupling of bone and cartilage degradation

Our data show that there is an uncoupling between degradation of bone and cartilage in metastatic breast and prostate cancer. Bone resorption increased in the group of patients that were in the initial phase of developing bone metastases having one to six bone metastases. Cartilage degradation was not elevated parallel to high bone resorption levels until at a late stage in the pathology of the cancer invasion involving multiple skeletal lesions. This disassociation of localized bone resorption and cartilage degradation in early stages of cancer patients with bone metastases suggests bone turnover alone is not the cause of the association of bone resorption and cartilage turnover.

Bone and cartilage turnover is coupled during some systemic pathologies such as postmenopausal osteoporosis and osteoarthritis. In response to estrogen supplementation or some anti-resorptives a reduction in both bone and cartilage degradation is observed, providing both a bone and cartilage sparring effect. We hypothesized that secondary to the increased bone resorption in case of patients having bone metastasis, an increased cartilage degradation would be observed additional to an even longer list of secondary complications and thereby reducing the quality of life. This was not the case and no treatment is needed for cartilage degradation. Thus, whereas systemic increased bone resorption resulted in secondary cartilage erosions, local increased bone turnover did not result in cartilage degradation.

Detection of bone metastases has been demonstrated for a number of biomarkers in prostate and breast cancer cohorts [15,10-21] in which it generally seems that collagenous markers are some of the best discriminators. However, no one has investigated cartilage degradation markers. Thus this is the first study to demonstrate that cartilage degradation does not correlate to number of bone metastases in prostate and breast cancer patients until patients reach a disease stage where they have more than 20 bone metastases. At this point, the progression of the disease is at a phase that might be aggressive enough to affect the joints of these patients or alternatively caused by a secondary affect from cancer treatment regimens or high expression of various cytokines by the cancer cells. However, these observations need further experimental data to be carefully investigated.

### Cathepsin K degradation of human bone

Cathepsin K is the most abundant collagenolytic protease of the osteoclast [22]. We investigated whether cathepsin K alone or the protolytic capacity of osteoclasts including cathepsin K, were capable of generating CTXII fragments in simple *in vitro *systems.

The *in vitro *data demonstrated that neither resorbing osteoclasts nor cathepsin K alone were able to generate CTXII fragments from human bone. In rats, collagen type II in present in some part of cortical bone, although the molar ratios of collagen type I to II remains to be investigated [9]. We used human cortical bone and clearly demonstrated with sensitive ELISA assays that CTXII cannot be released by cathepsin K alone or osteoclasts from bone. These findings either suggest that collagen type II is not present in human bone, or that cathepsin K cannot generate the CTX-II epitope. In addition, the vast proteolytic machinery of the osteoclast may destroy the CTX-II epitope if generated.

## Conclusion

Our data suggest that an uncoupling between bone and cartilage degradation occurs in these cancer patients with bone metastases. Thus the coupling observed in other disease groups can be disassociated and that cartilage degradation is not of concern for these patients. We clearly demonstrate that osteoclasts or cathepsin K alone are not able to generate CTXII fragments from human bone *in vitro*, suggesting that clinical CTX-II originate from cartilage.

## Competing interests

DJ. Leeming, I. Byrjalsen, P. Qvist, MG. Sørensen, C. Christiansen and MA. Karsdal are full-time employees of Nordic Bioscience involved in the development of biomarkers. C. Christiansen is as well a stock holder in Nordic Bioscience. The authors M. Koizumi, N. Lynnerup and M. Fregerslev declare that they have no competing interests

## Authors' contributions

DJL carried out the biochemical marker assessment, statistical calculations in collaboration with IB, and prepared the manuscript. IB carried out the statistical analysis in collaboration with DJL. MAK, PQ and CC carried out design of experiments ands assisted in all phases of the preparation of the manuscript. MK carried out the design of the study and collected samples at the Cancer Institute Hospital, Tokyo, Japan. NL and MF collected human bone samples from autopsies at the Forensic Science Institute, Copenhagen, Denmark. MGS carried out the osteoclast experiments. All authors read and approved the final manuscript.

## Pre-publication history

The pre-publication history for this paper can be accessed here:


